# The association between first trimester iron deficiency without anemia and the development of iron-deficiency anemia prior to childbirth

**DOI:** 10.1016/j.ajog.2026.03.012

**Published:** 2026-03-19

**Authors:** Nicole Konecke, Tracy L. Jackson, Irving Angeles, Iman I. Sigman, Crystal F. Ware, Ashley E. Benson, Joseph J. Shatzel, Nandini Raghuraman, Molly J. Stout, Ann M. Bruno, Steven Fein, Carolyn Webster, Michael Auerbach, Jamie O. Lo, Methodius G. Tuuli, Adam K. Lewkowitz

**Affiliations:** Department of Obstetrics and Gynecology, Warren Alpert Medical School at Brown University, Providence, RI; Department of Obstetrics and Gynecology, Warren Alpert Medical School at Brown University, Providence, RI; Department of Obstetrics and Gynecology, Warren Alpert Medical School at Brown University, Providence, RI; The Warren Alpert Medical School at Brown University, Providence, RI; Department of Obstetrics and Gynecology, Warren Alpert Medical School at Brown University, Providence, RI; Department of Obstetrics and Gynecology, Oregon Health & Science University, Portland, OR; Division of Hematology and Medical Oncology, Oregon Health & Science University, Portland, OR; Department of Biomedical Engineering, Oregon Health & Science University, Portland, OR; Department of Obstetrics and Gynecology, Washington University School of Medicine, St. Louis, MO; Department of Obstetrics and Gynecology, University of Michigan School of Medicine, Ann Arbor, MI; Department of Obstetrics and Gynecology, Division of Maternal-Fetal Medicine, University of Utah Health, Salt Lake City, UT; Heme On Call, Telemedicine-Based Hematology Practice, Miami, FL; Department of Obstetrics and Gynecology, Division of Maternal-Fetal Medicine, University of Alabama, Birmingham, AL; Department of Medicine, Georgetown School of Medicine, Washington, DC; Department of Obstetrics and Gynecology, Oregon Health & Science University, Portland, OR; Department of Urology, Oregon Health & Science University, Portland, OR; Department of Obstetrics and Gynecology, Warren Alpert Medical School at Brown University, Providence, RI; Department of Obstetrics and Gynecology, Warren Alpert Medical School at Brown University, Providence, RI; Center for Digital Health, Brown School of Public Health, Providence, RI

## OBJECTIVE:

To decrease the risk of adverse maternal and neonatal outcomes,^1,2^ iron therapy is recommended for pregnant people with iron deficiency anemia (IDA).^1^ To support our ongoing multicenter randomized trial on the optimal iron therapy for antenatal IDA (NCT05462704),^3^ we initiated universal ferritin screening, although this practice is not currently recommended.^4^ Nevertheless, perhaps because normal pregnancy physiology may cause first trimester iron deficiency without anemia (IDWA), defined as ferritin <30 ng/mL with hemoglobin ≥11 g/d, to progress to IDA,^5^ a clinical consensus based on expert opinion recommended treating iron deficiency in pregnancy regardless of anemia.^6^ Here, we examine the association between first trimester IDWA and the development of IDA later in pregnancy.

## METHODS:

Our pregnant patients receive a complete blood count and ferritin as part of their intake obstetric laboratory panel and are prescribed prenatal vitamins with iron. In this prospective cohort study, individuals with singleton first-trimester gestations who obtained an intake obstetric laboratory panel from February 2023 to July 2025 were included. Those with anemia were excluded. The primary outcome was IDA prior to childbirth, defined as being prescribed intravenous or oral iron therapy at any gestational age or progression to IDA before delivery. Secondary outcomes are described in Table 2. Relative risks were calculated adjusting for race and ethnicity. This study was institutional review board—approved.

## RESULTS:

Among 511 people, 46 (9%) had first trimester IDA and were excluded, while 129 (25%) had first trimester IDWA, and 336 (66%) were iron replete and nonanemic. Those with IDWA were more likely to be Hispanic or Native American than those who were iron replete and nonanemic despite similar age, parity, and body mass index (Table 1). After adjusting for race/ethnicity, those with first trimester IDWA were at nearly a 2-fold higher risk of developing IDA prior to childbirth (IDWA n=87 [67%] vs iron-replete, nonanemic n=118 [35%]; adjusted relative risk, 1.91; 95% confidence interval, 1.57—2.31). There was no difference in secondary outcomes (Table 2).

## CONCLUSION:

Universal screening for iron deficiency by adding ferritin to obstetric intake laboratories identified that first trimester IDWA affects one in 4 people in our clinics. Of those with first trimester IDWA, the majority (67%) developed IDA before childbirth, corresponding to nearly a 2-fold increased risk compared to those who were iron-replete, nonanemic in the first trimester. These findings demonstrate that first trimester IDWA has high likelihood of progressing to IDA, for which treatment is recommended.^1,2^ This study’s strengths included its prospective design, its diverse patient population, and low risk of loss to follow-up as everyone in our cohort received all perinatal care within our healthcare system. Limitations include excluding those screened for iron deficiency after the first trimester due to late initiation of prenatal care (although they may be at a higher risk for IDA^1^), completing the study in the United States (which limits generalizability to global settings), and that our analyses were not stratified by anemia severity. Nevertheless, these findings suggest that identifying IDWA in the first trimester provides an opportunity to prevent overt IDA and potential downstream perinatal morbidity.

## Supplementary Material

1

2

## Figures and Tables

**TABLE 1:** Patient demographic and obstetric characteristics, stratified by first trimester ferritin and hemoglobin results

Characteristics	No anemia, no iron deficiency (n=336)	Iron deficiency without anemia (n=129)	*P* value
Age <35 y	261 (78)	105 (81)	.5
Race			<.001
White	157 (47)	38 (29)	
Black	49 (15)	21 (16)	
Hispanic	16 (4.5)	19 (15)	
Asian	16 (4.8)	8 (6.2)	
Native American	6 (1.8)	1 (0.8)	
Multiracial	35 (11)	18 (14)	
Prepregnancy body mass index	30 (26, 34)	29 (25, 33)	.3
Federal health insurance	177 (53)	75 (58)	.6
Multiparity	333 (99)	129 (100)	.6

Data presented as n (%) or median (interquartile range).

**TABLE 2:** Perinatal outcomes, stratified by first trimester ferritin and hemoglobin results

Outcome	No anemia, no iron deficiency (n=336)	Iron deficiency without anemia (n=129)	*P* value	Relative risk (RR) (95% confidence interval [CI])	Adjusted RR (95% CI)
Maternal outcomes					
Iron deficiency anemia during pregnancy	118 (35)	87 (67)	<.001	1.92 (1.59, 2.31)	1.91 (1.57, 2.31)
Anemia during delivery hospitalization	52 (16)	31 (24)	0.045	1.50 (1.01, 2.23)	1.62 (1.09, 2.42)
Blood transfusion during delivery hospitalization	24 (7.3)	10 (7.9)	.8	–	–
Mode of delivery			.7	–	–
Spontaneous vaginal delivery	198 (60)	77 (60)		–	–
Operative vaginal delivery	11 (3)	6 (5)		–	–
Cesarean delivery	122 (36)	45 (35)		–	–
Screen positive for postpartum depression at 6 weeks postpartum	n=224 15 (7)	n=93 9 (10)	.4	–	–
Neonatal outcomes					
Preterm delivery	33 (10)	18 (14)	.2	–	–
Infant birthweight (grams)	3315 (2290, 3675)	3300 (3035, 3590)	.8	–	–
Neonatal intensive care unit admission	35 (11)	14 (11)	1.0	–	–
Perinatal morbidity^a^	1 (0.3)	3 (2)	.067	–	–

Data presented as n (%) or median (interquartile range). If adjusted, relative risks adjust for race and ethnicity.

a Defined as stillbirth, neonatal death within 72 hours of delivery, or neonatal death prior to discharge.

## AJOG at a Glance

### Why was this study conducted?

First trimester iron deficiency without anemia (IDWA) (ferritin <30 ng/mL with hemoglobin ≥11 g/dL) may progress to iron deficiency anemia (IDA), which is associated with adverse perinatal outcomes for mother and infant. We aimed to examine the association between first trimester IDWA and subsequent development of antenatal IDA.

### Key findings

Among 511 people, 129 (25%) had first trimester IDWA. Of these, 87 (67%) were diagnosed with IDA and/or were treated for IDA with iron therapy prior to delivery, compared with 35% (n=118 among those who were iron-replete, nonanemic).

### What does this add to what is known?

First trimester IDWA is common and associated with high likelihood of being diagnosed with and/or being treated for IDA during pregnancy. These findings suggest that identifying and treating IDWA in the first trimester may provide an opportunity to prevent IDA and associated morbidity.

## References

[1] Drukker L, Hants Y, Farkash R, Ruchlemer R, Samueloff A, Grisaru-Granovsky S. Iron deficiency anemia at admission for labor and delivery is associated with an increased risk for cesarean section and adverse maternal and neonatal outcomes. Transfusion 2015;55:2799–806. 26246160 10.1111/trf.13252

[2] Congdon EL, Westerlund A, Algarin CR, Iron deficiency in infancy is associated with altered neural correlates of recognition memory at 10 years. J Pediatr 2012;160:1027–33. 22244466 10.1016/j.jpeds.2011.12.011 PMC3360801

[3] Lewkowitz AK, Stout MJ, Carter EB, Protocol for a multicenter, double-blinded placebo-controlled randomized controlled trial comparing intravenous ferric derisomaltose to oral ferrous sulfate for the treatment of iron deficiency anemia in pregnancy: the IVIDA2 trial. Contemp Clin Trials 2022;123:106992. 36368479 10.1016/j.cct.2022.106992 PMC9729403

[4] Cantor AG, Holmes R, Bougatsos C, Atchison C, DeLoughery T, Chou R. Screening and supplementation for iron deficiency and iron deficiency anemia during pregnancy: updated evidence report and systematic review for the US preventive services task force. JAMA 2024;332:914–28. 39163033 10.1001/jama.2024.13546

[5] Benson AE, Lo JO, Caughey AB. Iron deficiency and iron deficiency anemia during pregnancy-opportunities to optimize perinatal health and health equity. JAMA Netw Open 2024;7:e2429151. 39163051 10.1001/jamanetworkopen.2024.29151 PMC11380762

[6] BensonAE, LoJO, AchebeMO, Management of iron deficiency in children, adults, and pregnant individuals: evidence-based and expert consensus recommendations. Lancet Haematol 2025;12:e376–88.40306833 10.1016/S2352-3026(25)00038-9

